# PQQ ameliorates D-galactose induced cognitive impairments by reducing glutamate neurotoxicity via the GSK-3β/Akt signaling pathway in mouse

**DOI:** 10.1038/s41598-018-26962-9

**Published:** 2018-06-11

**Authors:** Xing-qin Zhou, Zhi-wen Yao, Ying Peng, Shi-shi Mao, Dong Xu, Xiao-feng Qin, Rong-jun Zhang

**Affiliations:** 1 Key Laboratory of Nuclear Medicine, Ministry of Health, Jiangsu Key Laboratory of Molecular Nuclear Medicine, Jiangsu Institute of Nuclear Medicine, Wuxi 214063 Jiangsu Province, PR China; 2 Department of Neurology, Yangpu Hospital, Tongji University School of Medicine, 200090 Shanghai, PR China

## Abstract

Oxidative stress is known to be associated with various age-related diseases. D-galactose (D-gal) has been considered a senescent model which induces oxidative stress response resulting in memory dysfunction. Pyrroloquinoline quinone (PQQ) is a redox cofactor which is found in various foods. In our previous study, we found that PQQ may be converted into a derivative by binding with amino acid, which is beneficial to several pathological processes. In this study, we found a beneficial glutamate mixture which may diminish neurotoxicity by oxidative stress in D-gal induced mouse. Our results showed that PQQ may influence the generation of proinflammatory mediators, including cytokines and prostaglandins during aging process. D-gal-induced mouse showed increased MDA and ROS levels, and decreased T-AOC activities in the hippocampus, these changes were reversed by PQQ supplementation. Furthermore, PQQ statistically enhanced Superoxide Dismutase SOD2 mRNA expression. PQQ could ameliorate the memory deficits and neurotoxicity induced by D-gal via binding with excess glutamate, which provide a link between glutamate-mediated neurotoxicity, inflammation and oxidative stress. In addition, PQQ reduced the up-regulated expression of p-Akt by D-gal and maintained the activity of GSK-3β, resulting in a down-regulation of p-Tau level in hippocampus. PQQ modulated memory ability partly via Akt/GSK-3β pathway.

## Introduction

Aging is a complicated multifactorial process that results in a gradual slow decline in physiological function and the ability of an organism to survive. Oxidative damage has been implicated to be a major factor in the decline in physiologic function that occurs during the aging process^1,2^. Several studies have suggested that accumulation of reactive oxygen species (ROS) and elevated oxidative stress are associated with neuronal dysfunction in various age-related neurodegenerative disorders^3–5^. Mitochondria have been revealed as an important link between the age-related oxidative damage and the alterations in physiologic function associated with aging^6–8^. Furthermore, studies reported that oxidative damage to mitochondria would increase the generation of ROS, and lead to impaired cognition^6,9–11^. Oxidative damage stimulates the generation of free radicals and release high level of glutamate that may aggravate damage to brain cells^12,13^. Glutamate is the critical excitatory neurotransmitter in the central nervous system. However, excessive glutamate stimulation can produce ROS, which will contribute to neuronal damage^14–17^. In addition, the cumulate of Glutamate and continuous increase of ROS trigger neuroinflammatory pathways, increasing the chemokines, cytokines, and prostaglandins^18–23^. Moreover, reports demonstrated that glutamate decreased the expression of p-GSK-3β, which would increase GSK-3β activity during glutamate-induced neurotoxicity^24^. Pharmacological treatments, which inhibit GSK-3β, have been reported to reduce cognitive impairment in AD mice^25^.

D-galactose (D-gal) has been considered an artificial aging model which induces oxidative stress and inflammatory response resulting in memory and synaptic dysfunction^26–28^. Chronic systemic administration of D-gal in rodents has been extensively used as an animal model for brain aging in various anti-aging studies^29–31^. It has been reported that animals receiving chronic successive administration of D-gal (50–500 mg/kg) for 4–8 weeks experienced cognitive and memory dysfunction^24,25,30^. It has been reported that D-gal induced behavioral and neurochemical changes that could mimic many characteristics of the natural process of brain aging^26,32,33^. This model exhibits accelerated aging in tissues especially brain^34–38^. The brain is particularly vulnerable to oxidative damage because of its high oxygen demand, high unsaturated lipid content, and relative deficiency in anti-oxidative defense mechanisms. Thus, initial studies suggested that antioxidant therapy is crucial to prevent aging or age-related neurodegenerative disorders^39,40^.

Pyrroloquinoline quinone (PQQ) is a redox cofactor. It has been found in various foods, including vegetable and animal tissues^41–43^. PQQ not only serves to mediate redox reactions in the mitochondrial respiratory chain, but also plays a potential role of scavenging ROS and attenuating oxidative stress in mitochondria^44^. Accumulating evidence shows that PQQ can protect neurons against glutamate-induced damage by scavenging ROS^17^.

In the present study, we investigated that the protective effects of PQQ against D-gal induced oxidative stress, cognitive impairment, glutamate level and inflammation factors in mouse brain. We further explored the link between oxidative stress, neurotoxicity, neuroinflammation, and its potential contribution in aging related disease.

## Results

### Effects of PQQ on cognitive impairment induced by D-gal

#### Y-maze test

**Figure 1:**
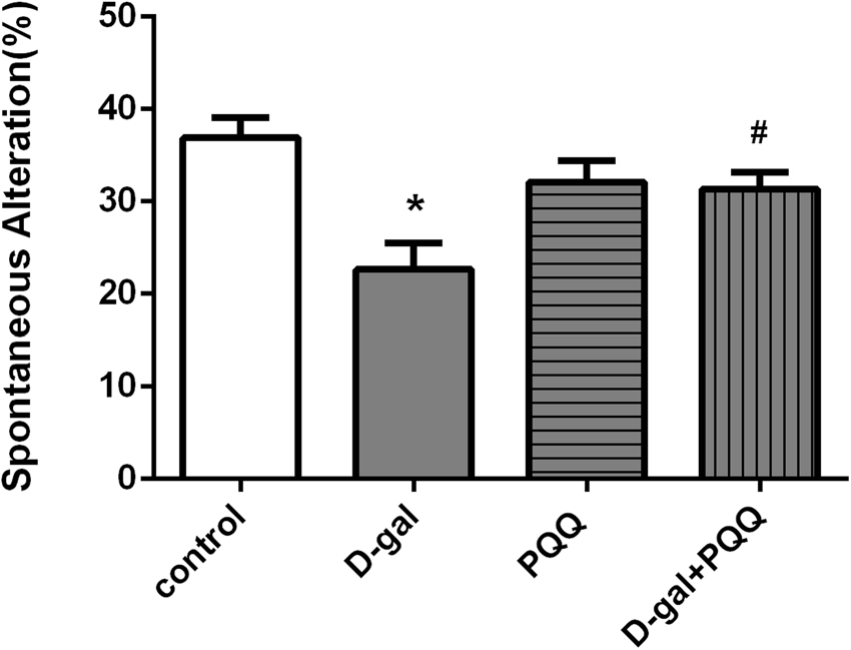
The percentage of spontaneous alteration in Y-maze of behavior test. The data are shown as a mean ± S.E.M. *P < 0.05 relative to control group. ^#^P < 0.05 relative to D-gal treated mice group.

The spatial working memory using spontaneous alteration behavior percentage (%) was analyzed using Y-maze task. Our results indicated that D-gal had a lower percentage of spontaneous alteration than control group, indicating less working memory (P < 0.05). However, the spontaneous alteration was significantly increased in mice who received PQQ either alone or in combination with D-gal (P < 0.05), indicating that PQQ improved memory in D-gal treated mice (Fig. 1).

#### Passive avoidance test

**Figure 2:**
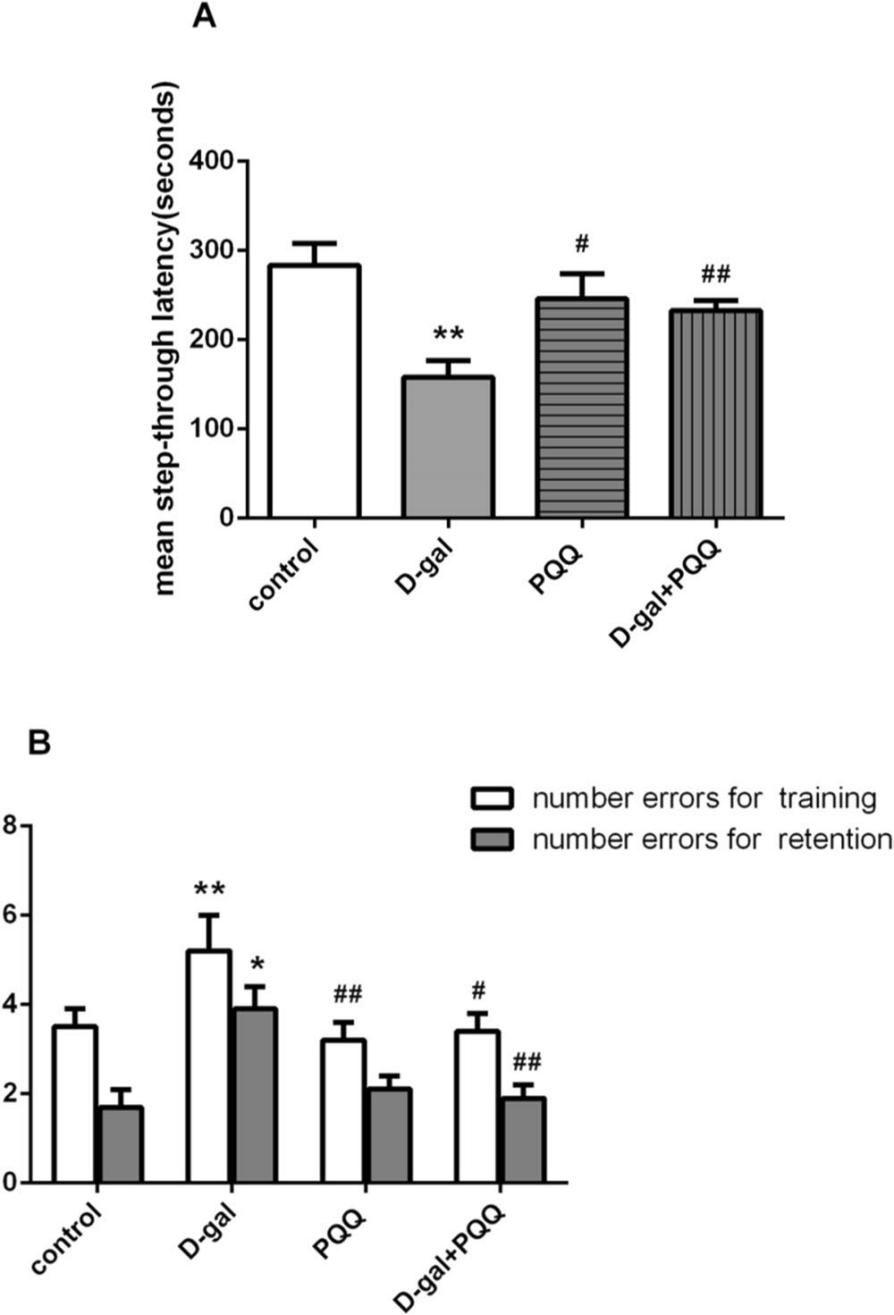
Effect of PQQ on learning and memory abilities of D-gal-treated mice in passive avoidance test. *P < 0.05, **P < 0.01 relative to control group. ^#^P < 0.05, ^##^P < 0.01 relative to D-gal group.

The results showed that PQQ significantly ameliorated cognitive ability of D-gal-treated mice for Passive avoidance test (Fig. 2). The model group mice had more error times and shorter latency for retention than those of control group mice (*P < 0.05, **P < 0.01 relative to control group, respectively). PQQ contributed to decreased number of mistakes (^#^P < 0.05, ^##^P < 0.01 relative to D-gal group, respectively) during the training session both alone and combining with D-gal. In the retention session, PQQ treatment with D-gal group mice showed longer latency and less mistakes compared with the model group mice (^##^P < 0.01 relative to D-gal group).

### PQQ ameliorated D-gal-induced oxidative stress and restored T-AOC activity in D-gal-induced aging mice

**Figure 3:**
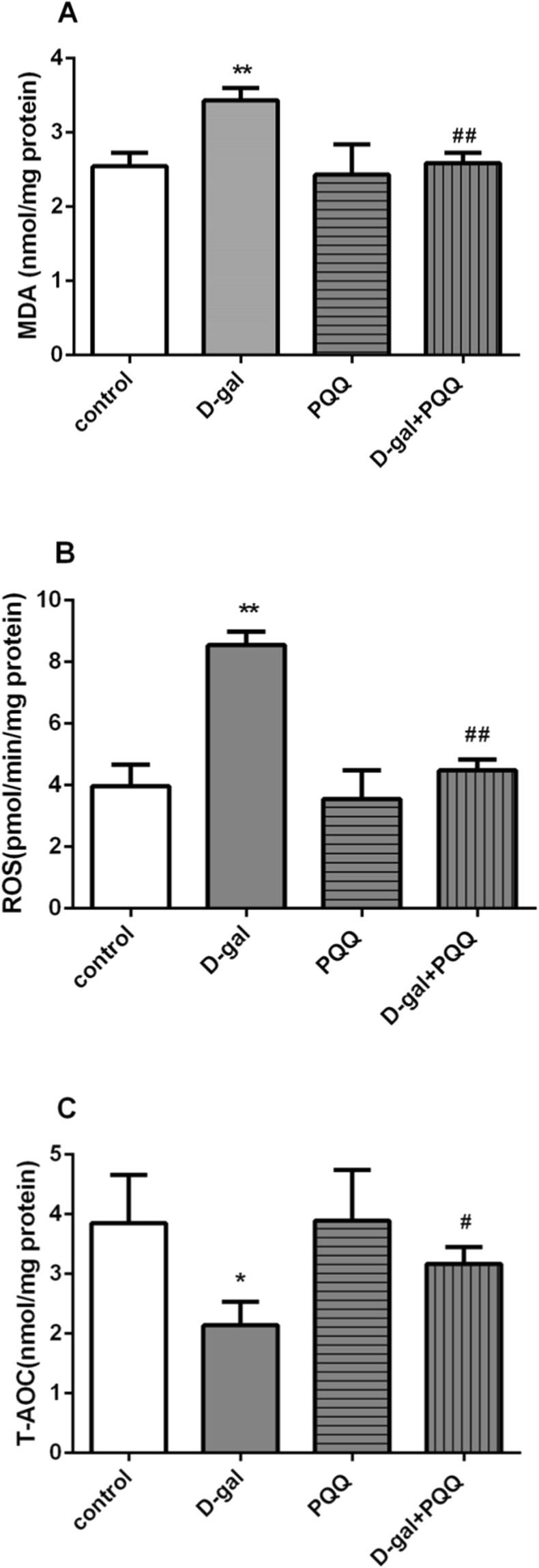
Effects of PQQ on MDA (**A**), ROS level (**B**) and T-AOC activities (**C**) in hippocampus of the D-gal-treated mice. *P < 0.05, **P < 0.01 relative to control group. ^#^P < 0.05, ^##^P < 0.01 relative to D-gal group.

Treatment with PQQ reduced the hippocampal MDA level, and thus reduced oxidative stress (Fig. 3A). In addition, the ROS levels were significantly higher in the hippocampal (p < 0.01) of D-gal-induced mice compared to the control mice. Treatment with PQQ inhibited the increase of levels of ROS in hippocampal (p < 0.01) (Fig. 3B). T-AOC activities were significantly lower in the hippocampal (p < 0.05) of D-gal-induced mice compared with normal control mice. Treatment with PQQ significantly ameliorated the reduction in hippocampal T-AOC activity (p < 0.05) (Fig. 3C). In addition, PQQ alone had no effect on MDA, ROS level or T-AOC activity in normal mice.

### Effect of PQQ on SOD expression in D-gal-induced mice

**Figure 4:**
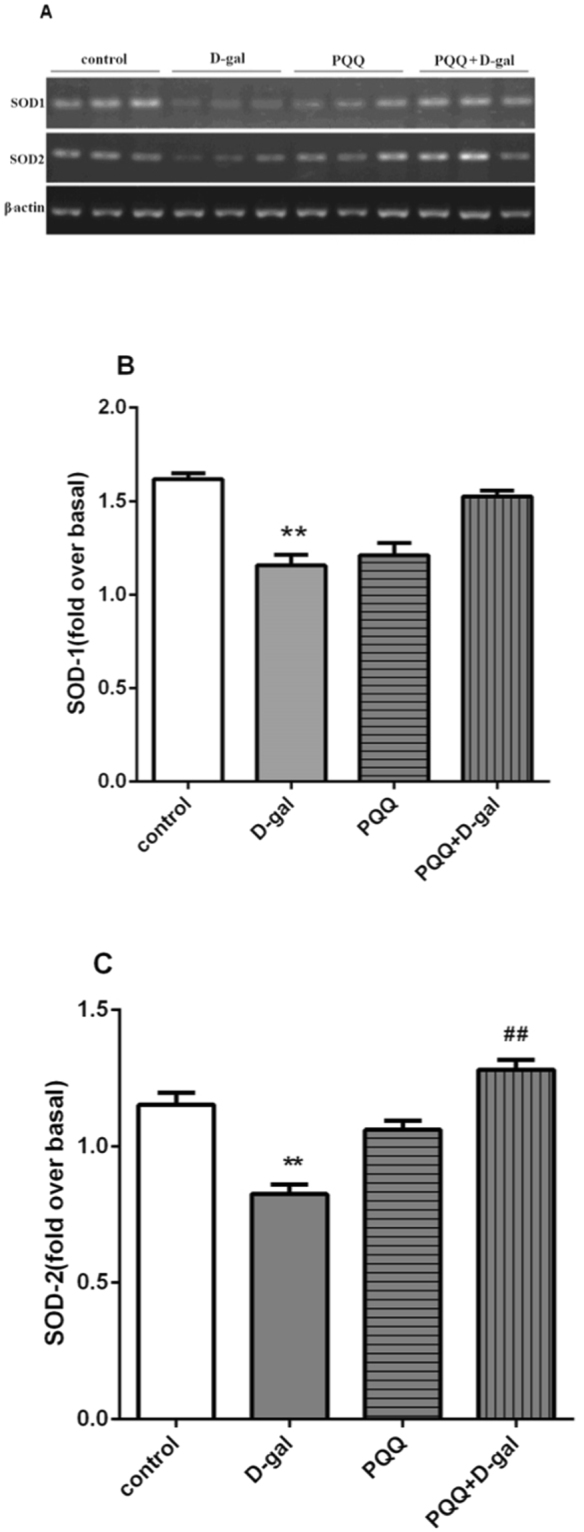
(**A**) RT-PCR analysis of superoxide dismutase (SOD) isoenzymes mRNA levels in the brain. (**B**,**C**) Densitometric analysis of gel images. Intensity of each band was quantified, normalized to the intensity of β-actin bands, and expressed as relative intensity; the results are shown as an average of seven subjects in each group. **P < 0.01 compared with control group. ^##^P < 0.01 compared with D-gal group.

Semiquantitative RT-PCR (Fig. 4A, Supplementary Info 2) was carried out to determine the levels of SOD1 and SOD2 expression. The mean levels of SOD1 mRNA and, to a greater extent, SOD2 mRNA were lower in D-gal mice than in the control group (P < 0.01) (Fig. 4). PQQ combine with D-gal treatment did not alter mRNA expression of SOD1 compared to the D-gal group, but statistically enhanced SOD2 expression (P < 0.01).

### Effect of PQQ on inflammatory factors include IL-2, IFN-γ levels and the production of Prostaglandin E2 (PGE2) in D-gal-induced mice

**Figure 5:**
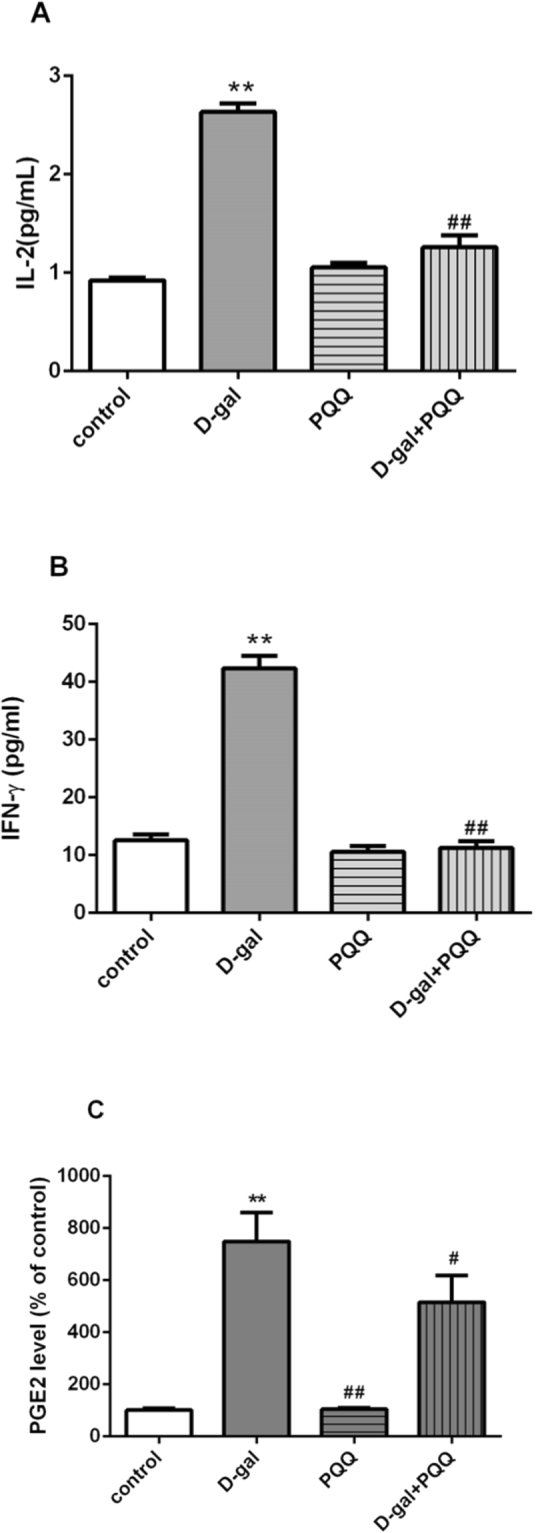
Effect of PQQ on IL-2 (**A**), IFN-γ (**B**) levels in serum and the production of PGE2 (**C**) in the hippocampus of the D-gal-treated mice. **P < 0.01, *P < 0.05 relative to control group; ^##^P < 0.01, ^#^P < 0.05 relative to D-gal group.

The effects of PQQ on IL-2 and IFN-γ levels in serum were detected by ELISA (Fig. 5A,B). D-gal treatment significantly increased IL-2 and IFN-γ levels 1.8- and 2.4-times (P < 0.01), respectively. Combining D-gal treatment with PQQ significantly decreased IL-2 from 2.63 ± 0.08 pg/ml in the D-gal group to 1.26 ± 0.12 pg/ml in the D-gal + PQQ group (P < 0.01). IFN-γ secretion was decreased from 42.35 ± 2.12 pg/ml in the D-gal group to 11.26 ± 1.18 pg/ml in the D-gal + PQQ group (P < 0.01). Figure 5C showed the effect of PQQ on the production of PGE2 in the hippocampus of D-gal-induced mice. As compared to control group, D-gal significantly increased the production of PGE2 to 6.41 times (P < 0.01). Treating the mice with PQQ significantly depressed the increase of production of PGE2 (p < 0.05), as compared to the D-gal-treated group. The production of PGE2 level in hippocampus were lower in PQQ treated mice than D-gal treated group (p < 0.01). These data indicate that PQQ reduced the enhanced IL-2 and IFN-γ caused by D-gal and regulated the production of PGE2.

### Effects of PQQ on glutamate content in D-gal-induced mice

**Figure 6:**
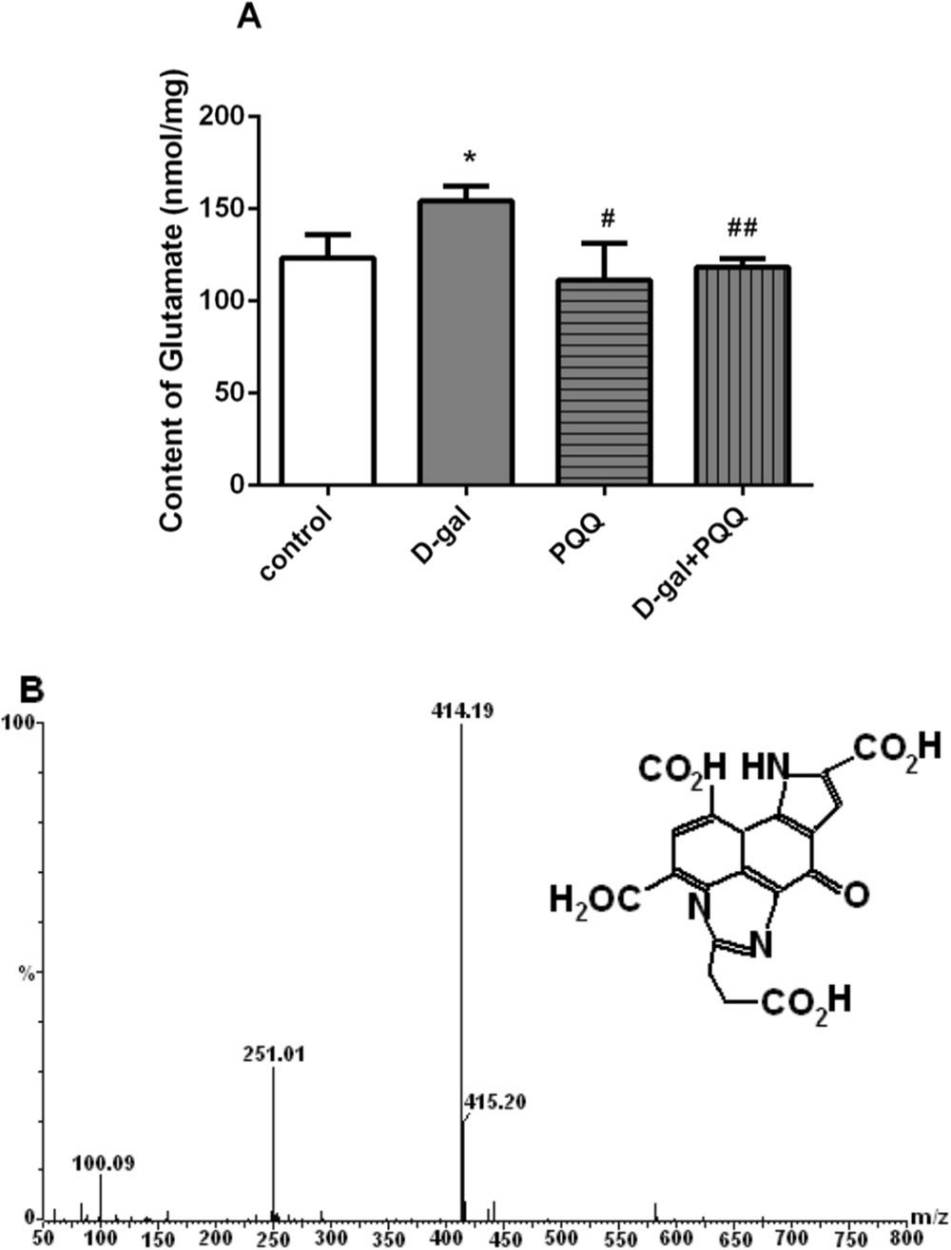
Effects of PQQ on glutamate content (**A**) in hippocampus of the D-gal-treated mice. *P < 0.05, **P < 0.01 relative to control group. ^#^P < 0.05, ^##^P < 0.01 relative to D-gal group. Negative-mode electrospray ionization mass spectra of production PQQ-glu (**B**) by UPLC.

Increased glutamate concentration in hippocampus were found in D-gal induced mice compared to control groups (25.14% vs p < 0.05). Only PQQ treated, the glutamate levels were not significant compared to control mouse. Glutamate level in hippocampus was lower in PQQ treated mouse than D-gal treated mouse (27.86% vs p < 0.05). The increased glutamate level in D-gal treatment were ameliorated significantly by PQQ treatment in hippocampus (23.32% vs p < 0.05) (Fig. 6A). Additionally, there was no significant difference in cortex and cerebellumin between these groups (Data not shown).

Furthermore, in molecular masses spectrum (Fig. 6B), the ions at m/z 414.19 ([M + H]+), 415.2([M + 2 H]+) was found in brain tissue of PQQ treatment group. We infer that the production has a molecular mass of 413 Da. These results are agreement with the previously reporters that PQQ can easily react with amino acids to form stable imidazolopyrroloquinoline^29,30^. Accordingly, the production with 413 Da molecular weight was tentatively identified as imidazolopyrroloquinoline PQQ-Glu (Fig. 6B). Given the evidence that imidazolopyrroloquinoline have redox and antioxidant activity as well as growth-promoting properties, a clear demonstration that complexes PQQ binding with amino acids exist in tissues has considerable importance. Further study on the beneficial effects of PQQ-Glu in neural function is necessary.

### Effect of PQQ on expressions of Akt/GSK3β signaling markers

**Figure 7:**
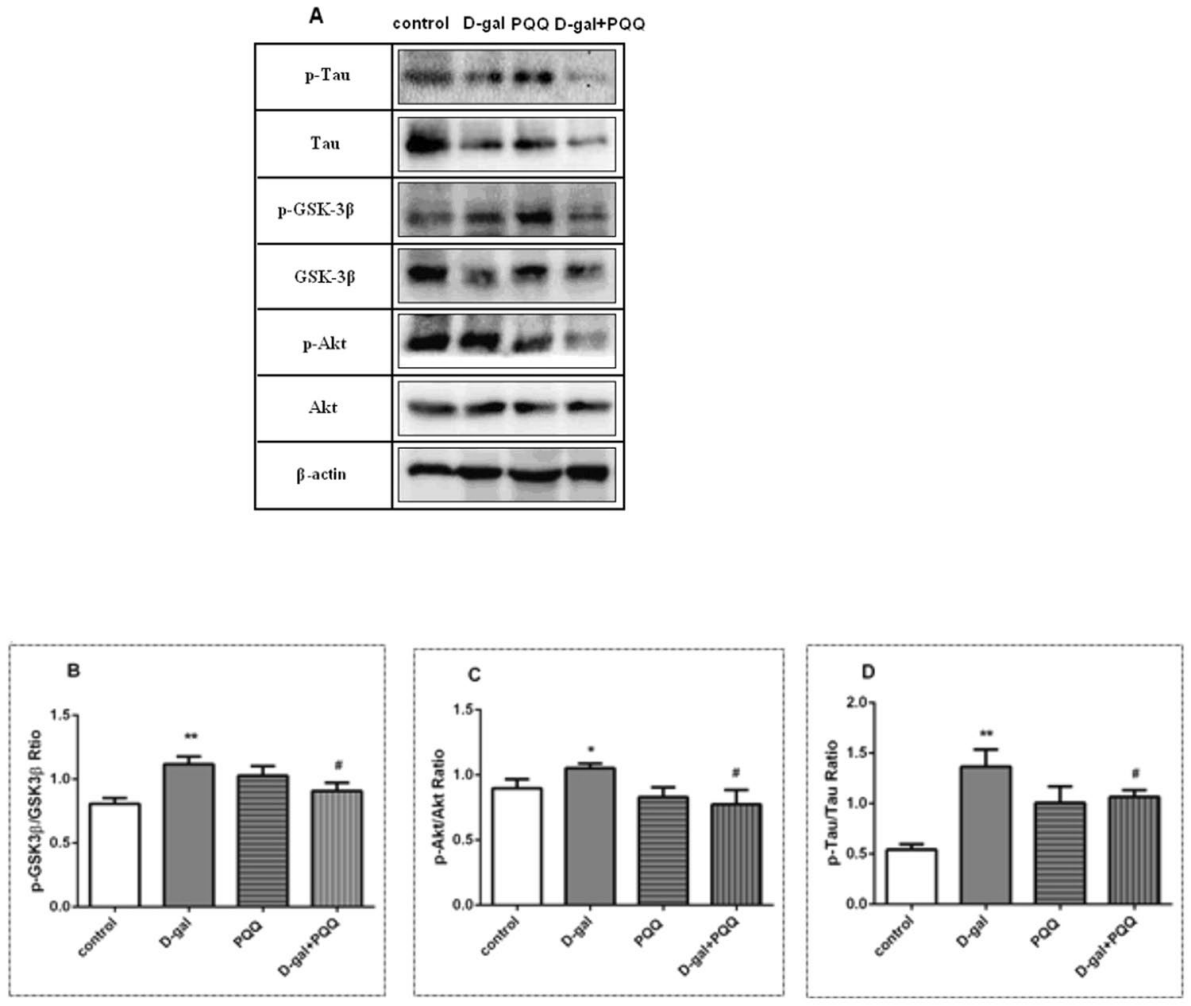
Western blot (**A**) analysis the effects of PQQ on GSK-3β (**B**), Akt (**C**) and Tau (**D**) phosphorylation in the hippocampus of the D-gal-treated mice. *P < 0.05, **P < 0.01 relative to control group. ^#^P < 0.05, ^##^P < 0.01 relative to D-gal group.

Western blot analysis (Fig. 7A, Supplementary Info 1) was used to assess the effect of PQQ on the Akt/GSK3β pathway in hippocampus subjected to D-gal. The results show that D-gal had no significant effect on the overall expression of Tau in hippocampus (p > 0.05). But mice treated with D-gal exhibited significant stimulation in the expression of p-Tau in hippocampus (p < 0.01), whereas the expressions of p-Tau in hippocampus were significantly attenuated by co-treatment with PQQ (p < 0.05, Fig. 7D). Although PQQ failed to modulate the expression of Akt in hippocampus, it was able to reduce the p-Akt expression in D-gal treated groups so as to decrease the ratio of p-Akt/Akt from 1.05 to 0.77 (p < 0.05, Fig. 7C). Likewise, PQQ was able to reduce the ratio of p-GSK-3β/GSK-3β from 1.11 to 0.91, which was beneficial to regulate the expression of GSK-3β during D-gal-induced aging (p < 0.05, Fig. 7A,B). However, there were no significant differences in cortex and cerebellumin in these groups (Data not shown).

## Discussion

The present study aimed to investigate the protective effect of PQQ on cognitive impairment in the aging mouse induced by D-gal and the possible underlying mechanisms.

The chronic administration of D-gal induces changes that resemble natural aging in animals, such as cognitive dysfunction, oxidative stress and neurodegradation. D-gal induced senescence acceleration has been accepted as a rodent model for use in research on aging studies^45,46^. D-gal can cause the accumulation of ROS *in vivo*, ultimately result in oxidative stress^37^. In addition, oxidative stress contributes to a chronic inflammatory process during aging and causes age-associated diseases^22,47^. It is well known that glutamate is the critical excitatory neurotransmitter in the central nervous system. Oxidative stress increases the generation of ROS and release excess glutamate^48,49^. Excess glutamate stimulation is a common pathway in brain injuries and degenerative diseases, and ROS production is usually considered to be involved in glutamate stimulation^50,51^. Furthermore, oxidative injury, excitotoxicity and inflammatory seem to be three important factors involved in process of aging.

Some studies had already confirmed that PQQ prevents oxidative damage in the brain and reduces the cognitive deficit caused by oxidative stress in rats during aging^52^. In our previous study, we found that PQQ may be converted into a derivative by binding with amino acid, which is beneficial to several pathological processes^53,54^. However, which derivative promotes antioxidant in aging brain and the underlying mechanism is yet unclear. For the first time, we found a beneficial glutamate mixture which may diminish neurotoxicity by oxidative stress. As well, we will discuss the relevant aspects concerning possible neuroprotection of PQQ in the oxidative stress and neuroinflammatory changes observed in aging.

### Antioxidation

The evidence showed that PQQ treatment can affect learning ability and memory function during oxidative stress in rats^55^. It is well known that PQQ is a well-characterized, free and redox cycling planar orthoquinone. Similar to other polyphenolic biofactors, there is strong evidence PQQ may play an important role in pathways to antioxidant potential^56^. Our results showed D-gal seriously impaired the learning and memory abilities. We found that this behavioral impairment was reversed when D-gal treated mice were simultaneously administered PQQ for the duration of the treatment (Fig. 1). The results are supported by previous studies, using MK-801 treatment in mice^54^ and hypoxia treatment in rats^55^, which proved that PQQ would prevent the cognitive deficit resulting from the oxidative stress.

We also observed that oxidative damage occurred in the D-gal induced aging mouse brain, and this damage was reversed by PQQ intervention. The consequences of oxidative stress can be measured by markers of damage including ROS, MDA and T-AOC. It has been suggested that accumulation of ROS induces severe damage to neuronal system. An increase in MDA is another important marker for lipid peroxidation^37^, which is a well-known indicator of oxidative damage of membranes under conditions of oxidative stress. In addition, total antioxidant capacity (T-AOC), as a non-enzymatic antioxidant, also indirectly reflects the level of oxidative stress^57^. According to our researches, PQQ decreased MDA (Fig. 3A) levels, and reduced ROS in D-gal aging model (Fig. 3B). Furthermore, in our T-AOC test, PQQ could renew the activity of T-AOC in the brain of D-gal-induced aging mice (Fig. 3C).

Apart from amelioration of MDA and ROS, PQQ also increased the activity of SOD, a major mitochondrial antioxidant enzyme that converts toxic superoxide to hydrogen peroxide. SOD is the first and the most important line of antioxidant enzyme defense against ROS protecting cells and tissues^58^. SOD2 null mice have been known to have short survival. B-Sod2−/− showed brain lipid peroxidation significantly increased, causing severe growth retardation^59^. Further more, mitochondrial SOD2 is thought to play an important role in cellular defense against oxidative damage by ROS^60^.

In this study, PQQ statistically enhanced SOD2 mRNA expression compared to the D-gal group (Fig. 4). Our findings provide direct supportive experimental evidence supporting that PQQ might have anti-aging effects due to its antioxidant role.

### Anti-inflammatory

In addition, the development of neuroinflammation has also been related to cognitive changes in aging related diseases^61–63^. Neuroinflammation in aging is characterized by the accumulation of many cytokines, in which the most obvious change is IL-2 production^64^. IL-2 and IFN-γ are produced by activated T cells, and aging disorders have been related to these cytokines exceedingly. Furthermore, chronic hypobaric hypoxia prompts the release of cytokines/chemokines viz. IFN-γ and IL-2 activating the signal transduction pathway related to oxidative stress^65^. In this study, the production of important cytokines such as IL-2 and IFN-γ was also investigated. D-gal increased the levels of the pro-inflammatory cytokines IL-2 and IFN-γ. PQQ decreased cytokines IL-2 (52.1%), and IFN-γ (73.4%) from D-gal treatment mice (Fig. 5). The results are agreed with the report that PQQ deprivation bring about decline immune function^66^. This study indicated that PQQ markedly reduce the inflammation in D-gal-treated brain, and thus protect the nervous system against oxidative damage.

More over, prostaglandins are major components of the neuroinflammatory process. Prostaglandin E2 (PGE2), a product of oxidative stress, is one of the most reliable biomarkers of lipid peroxidation in the human body, and of aging related diseases^67,68^.

It has been reported that prostaglandin levels are higher in the brains of AD patients than in control brains^69,70^. Our results showed that hippocampus PGE2 was significantly increased in D-gal treated mouse. Treatment with PQQ suppressed hippocampus level of PGE2. As the levels of pro-inflammatory prostaglandins increase, the capability of clearance must also be improved to maintain low level prostaglandin and prevent additional inflammation. Our results suggest that PQQ may influence the generation of proinflammatory mediators, including cytokines and prostaglandins during in aging process.

### Anti-excitotoxicity

Glutamate is the main excitatory neurotransmitter in the central nervous system (CNS) and the toxicity of glutamate has been shown to induce neuronal cell death through oxidative stress^71^. In mammalian brain, Glu is by far the most prevalent neurotransmitter, which is excitatory working on over 90% of the synapses in the brain^72^. It is considered to be the major mediator of excitatory signals and is probably involved in cognitive domain of the central nervous system^73^. Glutamatergic neurotransmission has already been adopted as a promising target for neuro disorder drug development. Furthermore, Liang *et al*. reported that reduction of the SOD2 level increased the levels of glutamate at the synapse and led to pathological conditions due to the downregulation of glutamate transporter 1 that clear glutamate from synapse^74^. In the brains of B-Sod2−/−, the potently downregulated expression of GLT-1 could induce a resultant increase in the glutamate concentration at synapse, leading to excitotoxicity. Therefore, it is of critical importance that the extracellular glutamate concentration must be kept enough low due to the toxicity of glutamate in high concentrations. It should be revealed that the distribution of glutamate in brain is a dynamic equilibrium with a rapid turnover in normal condition. Extracellular glutamate levels are maintained through a balance between release and uptake in the normal brain function. Abnormal glutamate uptake has been shown to be involved in the pathogenesis of neurological disorders where glutamate neurotoxicity plays a major role. Glutamate uptake is one of the main mechanisms responsible for keeping glutamate concentration at low level to resistant glutamate toxicity in normal intact brain tissue for the long-term^75^. However, there is no match to the capabilities of glutamate uptake in abnormal condition. The previous research was focused on the ability of glutamate transporter function for promoting protective effects against glutamate excitotoxicity to neurons. It has been proved that glutamate bound to various proteins is likely to reduce the concentration of free glutamate in glutamate transport cycles^76^. Inspired by the successful studies of binding with proteins, it seemed tempting to look for effective compounds binding to glutamate for the normal brain function. It is well known that PQQ has special chemical structure and characteristics and easy to combine with different substances^42^. Our previous studies found that PQQ was easily converted into derivatives with neurotransmitters amino acid which have beneficial capabilities to several physiological processes^53,77^. The concentrations of amino acid in the central nervous system tissue vary according to the physiological state. From Fig. 6A, it can be seen that the concentrations of glutamate in D-gal-induced mice were significantly higher than those in control group. D-gal is a reducing sugar having affinity for free amines of amino acid in proteins which lead to accumulation of advanced glycation end products, subsequently lead to elevated oxidative stress^78^. Because of the competitive interaction with free amines of amino acid, the potential efficacy of PQQ in inhibiting the production of glycation end products by binding with amino acid, and prevented the oxidative stress. Moreover, the “bingding” reduced excitability toxicity by decreasing the content of glutamate. From Fig. 6B, new production was found and identified in the brain of PQQ treated mouse compared with untreated ones by UPLC/MS. The results indicated that the complex may be beneficial for anti-aging effects of PQQ, which were related to the action of mechanism. PQQ inhibits excitotoxicity by which it may produce complex via binding with free glutamate in the brain, providing new insights into future research of central nervous system and drug discovery.

### Akt/GSK-3β signaling

Glycogen Synthase Kinase-3β (GSK-3β) is a protein kinase involved in memory formation which impairs memory through excessive phosphorylation of substrates, such as tau protein^79,80^. GSK-3β has been revealed to provide an opportunistic target for learning and memory related disorders in aging process^81^. Several studies have demonstrated the link between GSK-3β and the neuropathology of AD^82,83^. Sintoni also reported that GSK-3β dysregulation in the hippocampus play a key role in memory impaired rat models^84^. In addition, the regulation of transcription process by GSK-3β is an important survival pathway against oxidative stress^85^. Pharmacological treatments, which inhibit GSK-3β, have been reported to repair cognitive impairment in AD mice^25^. GSK-3β acts as a downstream regulator, and its activity is mainly regulated by phosphorylation or dephosphorylation via signaling pathways^86^. In addition, decreased the expression of p-GSK-3β was also reported during glutamate-induced neurotoxicity^24^. It has been documented that protein kinase B (Akt) is a major kinases to inactivate GSK-3βthrough phosphorylation^87^. The high enzymatic activity is inhibited upon phosphorylation on residue ser9 of GSK-3β by Akt^88^. Therefore, drugs that are able to down-regulate the activity of GSK-3β hold high promise of therapeutic effects for aging-related diseases.

Obviously, PQQ has been proved to attenuate oxidative stress in mitochondria^44,89^, and antagonize glutamate-induced neuronal injury both *in vitro* and *in vivo*^17,90,91^. It has been observed that phosphorylated Akt was regulated by PQQ treatment in glutamate-injured hippocampal neurons^17^. The molecular mechanisms underlying these effects have not been fully elucidated. Although it was well proposed that PQQ might have antioxidant role, the direct supportive experimental evidences linking the mechanism of action with anti-aging have rarely been reported so far. The biological effects of PQQ have been intensively studied, but it is not clear whether the neuroprotective effects of PQQ against oxidative stress is mediated through reducing glutamate and suppressing neuroinflammatory mediators by Akt/GSK-3β signal pathway interruption.

In this experiment, we found D-gal up-regulated the expression of p-AKT in the hippocampus, the active form of AKT, leading to decrease the activity of GSK-3β. Fortunately, the expression of p-Akt was markedly reduced and maintained the activity of GSK-3β by PQQ, resulting in an down-regulation of p-Tau level in hippocampus. Moreover, our present study indicated that PQQ prevents cognitive impairment of D-gal induced mouse. Interestingly, PQQ reduced the phosphorylation of Tau and maintained the activity of GSK-3β, which was essential for memory and learning in aging process. In addition, the inhibition of MDA activity and ROS production might be involved in the anti-oxidative stress effect of PQQ. Its mechanism may be related to the down-regulation the expression of SOD2. The decline of SOD2 activity can lead to the disruption of oxidative stress balance, which damages mitochondrial function and causes aging related diseases. This effect is likely related to the reduction of oxidative stress via the Akt/GSK-3β pathway. Therefore we inferred that PQQ modulated memory ability partly via Akt/GSK-3β pathway. Our results showed that antioxidant capability of PQQ might be mediated by the inactivation of GSK-3β in an increase of p-GSK-3β. Furthermore, for the first time, we revealed that PQQ could ameliorate the memory deficits and neurotoxicity induced by D-gal via binding with excess glutamate, inhibiting oxidative stress, and eliminating inflammation through GSK-3β/Akt signaling pathway. This is in agreement with the evidence that PQQ protected cultured hippocampal neurons against glutamate excitotoxicity^90,91^

In conclusion, our findings demonstrate that PQQ effectively improves D-gal-induced cognitive dysfunction provide a link between Glu-mediated neurotoxicity, inflammation and oxidative stress. Taken together, our results suggest that PQQ is beneficial in preventing cognitive deficits during aging process. More work remains to be done before we elucidate these mechanisms.

## Materials and Methods

### Reagents and drugs

D-gal (Sigma-Aldrich, USA) and PQQ (Shanghai Med. Co., China) solutions were freshly prepared in sterile water. MDA (catalog no. S0131) were purchased from Beyotime Institute of Biotechnology (China). T-AOC (catalog no. S0116, Beyotime Institute of Biotechnology, China). IL-2 and IFN-γ production were measured using ELISA Ready-SET-Go! ® kits (eBioscience, USA). PGE2 were tested using PGE2 EIA kit (Cayman Chemical Co., Ann Arbor, MI, USA). Total RNA Extraction Reagent was purchased from Beyozol (China); High-Capacity cDNA Reverse Transcription Kits were purchased from Applied Biosystems (China). Semiquantitative RT-PCR was performed using primers designed by and obtained from Sangon Biotech, LTD (China). Glu were purchased from ACROS (Belgium, USA). Methanol was of HPLC grade and obtained from CNW Technologies GmbH (Hanau, Germany).

### Animals and treatment

ICR mice were supplied from Cavens Lab Animal Co. (Changzhou, China). The mice were aged 3 months old, weighed 32 ± 2 g. Animals were housed at a temperature of 25 ± 1 °C and relative humidity of 55%–60% with a controlled light-dark cycle. The mice had free access to food and water. The mice were divided into four groups of 10 male mice at random. Two groups of mice received daily subcutaneous injection of D-gal at dose of 150 mg·kg^−1^ · d^−1^ for 6 weeks, and the third group of mice served as normal control was injected with saline (0.9% NaCl) only. Meanwhile, one group of D-gal-treated mice received PQQ at a dose of 100 μg · kg^−1^ · d^−1^. At the same time, the other group of D-gal-treated mice and the control group of mice were given distilled water, and the fourth group received PQQ (100 μg·kg^−1^ · d^−1^) only. The dosages and pretreatment duration for the compounds were used based on the previous studies^56–58^. All experiments were approved by the Animal Care and Ethics Committee of Jiangsu Institute of Nuclear Medicine and carried out according to the National Institutes of Health Guidelines for the Care and Use of Laboratory Animals.

### Behavioral tests

#### Y-Maze Test

Y-maze constructed from black painted wood with three arms of 50 cm long, 20 cm high, and 10 cm wide at the bottom and at the top was used for behavioral analysis. The mice were placed at the center and were allowed to move freely for three 8-min sessions. The test was performed in a sound-isolated and dark room. The arm entries series was noted for observation. Spontaneous alteration was defined as the successive entry of the mice into the three arms in overlapping triplet sets. Alteration behavior (%) was calculated as [successive triplet sets(entries into three different arms consecutively)/total number of arms entries −2] × 100^59^.

### Passive avoidance test

The passive avoidance apparatus contains two chambers (20 × 25 × 30 cm each) of equal size, one is an illuminated by a 4 W fluorescent lamp and other is a dark one. Both of these compartments are linked with a door which allows mice to cross freely from one compartment to another. These compartments contain a grid floor that consists of rods having a distance of 0.5 cm between the rods. Initially the animals were trained by placing them in the illuminated compartment, and an electric shock (40 V, 0.5 A, 1 s) was delivered to their paws via the rods of the grid floor, causing them to move into the dark chamber. Mice were then immediately removed and returned to their home cages. During the retention trial that occurred 24 h later, mice were placed in the illuminated chamber, and the latency to enter the dark chamber was recorded (i.e., step-through latency). In this session, the number of repeated step-down in 300 s was counted as errors.

### Measurement of ROS, MDA levels, and total antioxidant capacity (T-AOC)

MDA, ROS, and T-AOC, were determined by using commercially available kits. ROS was measured as described previously with some modification, based on oxidation of 2,7-dichlorodihydrofluorescein diacetate (DCFH-DA) to 2,7-dichlorodihydrofluorescein^60^. The data are expressed as picomole DCF formed per minute per milligram protein. The thiobarbituric acid reaction method was used to determine MDA content. MDA reacts with thiobarbituric acid to form a stable chromophoric molecule that can be measured at the wavelength of 532 nm^61^. MDA content was expressed as nanomoles per milligram of tissue protein. The Ferric reducing power (FRAP) test method was used to quantify the T-AOC of the organs^62^. After mixing the FRAP reagent with the organ sample, the absorbance was determined every 1 min for a total of 10 min. Fe (II) standard solution was tested in parallel.

### RNA extraction and RT-PCR analysis

T RNA was extracted from brain using the kit as directed by the manufacturer’s protocol. Using the High-Capacity cDNA Reverse Transcription Kits, 1.5 μg of RNA was transcribed into cDNA according to the manufacturer’s instructions, and cDNA samples were stored at −70 °C. Semiquantitative RT-PCR was performed using the primers obtained from Sangon Biotech, LTD (China). The sequences of the primers are designed as follows: SOD1(352 bp), Forward 5′-ACCATCCACTTCGAGCAG-3′, Reverse 5′-TTTCTTCATTTCCACCTTTG-3′; SOD2(396 bp), Forward, 5′-GCACCACAGCAAGCACC-3′, Reverse, 5′-CCCAGCAGCGGAATAAG-3′; β-actin(384 bp), Forward, 5′-GGGAAATCGTGCGTGACAT-3′, Reverse, 5′-CAGGAGGAGCAATGATCTT-3′.

### ELISA analysis for IL-2, IFN-γ and PGE2

IL-2 and IFN-γ production were measured using ELISA Ready-SET-Go! ® kits with sensitivity of 2 and 4 pg/ml, respectively as described by the manufacturer. The absorbance of the reaction was measured at 450 nm with subtraction of background at 570 nm using a microplate reader. PGE2 concentrations were measured using a commercially available PGE2 EIA kit according to manufacturer’s instructions as the previous described^31^.

### Western blot analysis

The Western blot assay was performed as previously described^24,88^. Briefly, the hippocampus was homogenized in lysis buffer (Beyotime Biotechnology, Haimen, China) and centrifuged at 12,000 rpm for 10 min at 4 °C. The concentration of hippocampal protein was measured using BCA assay kit (Sangon, Shanghai, China). The dilution of primary antibodies as follows: β-actin (1:4000, Abcam, UK); GSK-3β; p-GSK-3β; AKT; p-AKT; Tau, p-Tau (1:2000, Cell Signaling Technology, USA). Each membrane was rinsed three times for 15 min and incubated with the secondary antibodies (1:8000, Abcam, UK). β-actin was used as the loading reference for data analysis.

### Measurement of glutamate

The levels of glutamate in brain tissues were analyzed using HPLC system as the our confirmed method. Furthermore, a related marker for the protection of glutamate neurotoxicity in hippocampus has been explored by UPLC/MS. The brain tissues were homogenized in ice-cold 0.5 M formic acid (5 mL/g) and the mixtures were centrifuged 15,000 g for 30 min at 4 °C. The supernatant was collected and run through solid-phase extraction columns (Styre Screen® H2P) with the aid of vacuum. Extraction cartridges were pretreated by rinsing with 1 ml of ethanol, followed by 1 ml deionized water contain 0.1% trifluoroacetic acid (TFA). After the samples were loaded onto columns, the columns were washed with 1 ml deionized water, followed by 2 ml of ethanol/o.1%TFA (60/40) in water. Eluants were evaporated to dryness by vacuum freeze-drying and waiting for analysis. The content of glutamate were determinated using UPLC-MS^49^. Molecular masses were carried out on a Waters Acquity ultraperformance liquid chromatography system (Waters, MA, USA) with a SQ Detector 2 mass spectrometer equipped with diode-array detector. Data were collected and processed with Waters MassLynx. A Waters BEH C18 column (2.1 × 100 mm, 1.7 um) was applied for reverse phase chromatographic separation. The optimized MS parameterswere set as follows: Desolvation temperature 400 °C, desolvation gas flow 600 L/h, source temperature 110 °C, cone gas flow 50 L/h, capillary voltage 3000 V, cone voltage 30 V. The initial mobilephase composition was held at 5/95 ACN/water contain 0.1% TFA (v/v) for 5 min followed by a linear gradient to 75:25 ACN:water contain 0.1% TFA (v/v) at 25 min.

### Statistical analysis

All statistical tests were performed with the SPSS v16.0 statistical software. One way analysis of variance was applied using Tukey’s posthoc comparisons. The data were expressed as mean ± SD of triplicate experiments. P-values below 0.05 were considered statistically significant.

## Electronic supplementary material

Supplementary Info 1

Supplementary Info 2
