# Supplementary material for: PQQ ameliorates D-galactose induced cognitive impairments by reducing glutamate neurotoxicity via the GSK-3β/Akt signaling pathway in mouse
Source: Sci Rep. 2018 Jun 11;8:8894. doi: 10.1038/s41598-018-26962-9 (PMC5995849; doi:10.1038/s41598-018-26962-9)
Supplement: Supplementary file 1 — Supplementary Info 1 [file 41598_2018_26962_MOESM1_ESM.pdf]

# PQQ ameliorates D-galactose induced cognitive impairments by reducing glutamate neurotoxicity via the GSK-3β/Akt signaling pathway in mouse

Xing-qin Zhou, Zhi-wen Yao, Ying Peng, Shi-shi Mao, Dong Xu, Xiao-feng Qin, Rong-jun Zhang

Fig. 7 (A)Western blot. (Original)

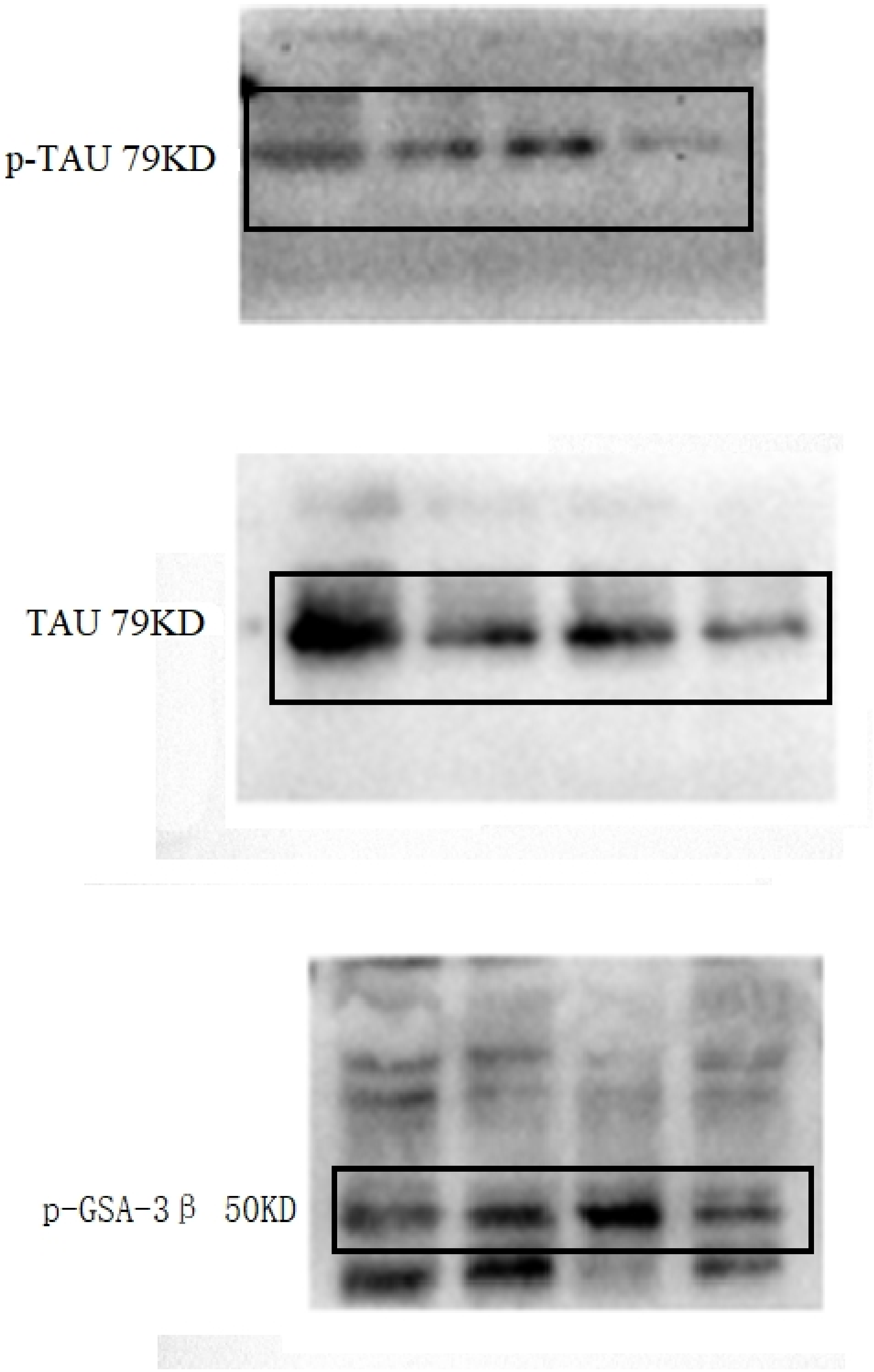

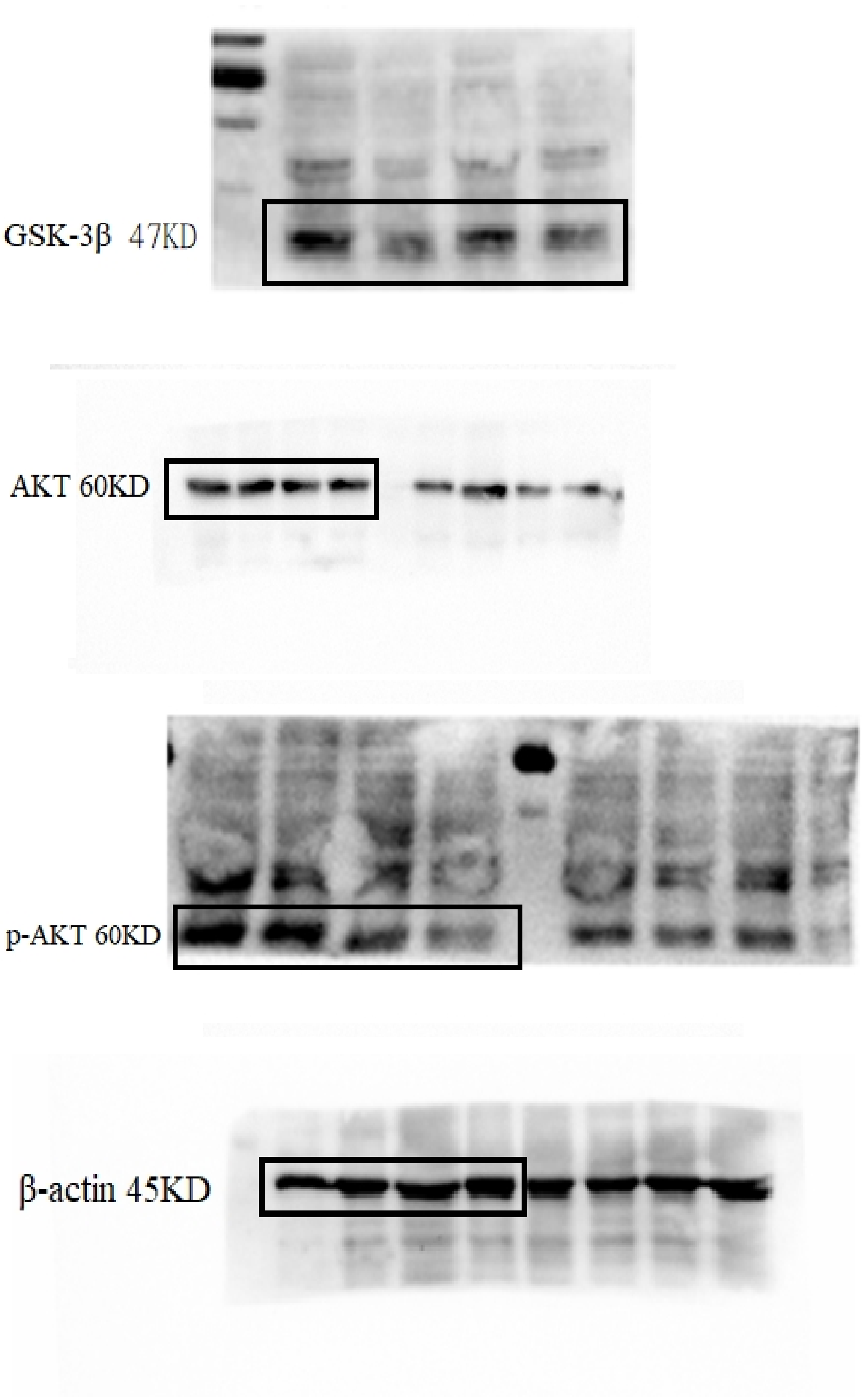
GSK-3β 47KD
AKT 60KD
p-AKT 60KD
β-actin 45KD
