# Supplementary material for: PQQ ameliorates D-galactose induced cognitive impairments by reducing glutamate neurotoxicity via the GSK-3β/Akt signaling pathway in mouse
Source: Sci Rep. 2018 Jun 11;8:8894. doi: 10.1038/s41598-018-26962-9 (PMC5995849; doi:10.1038/s41598-018-26962-9)
Supplement: Supplementary file 2 — Supplementary Info 2 [file 41598_2018_26962_MOESM2_ESM.pdf]

PQQ ameliorates D-galactose induced cognitive impairments by reducing glutamate neurotoxicity via the GSK-3β/Akt signaling pathway in mouse

Xing-qin Zhou, Zhi-wen Yao, Ying Peng, Shi-shi Mao, Dong Xu, Xiao-feng Qin, Rong-jun Zhang

Fig. 4 (A) RT-PCR analysis of superoxide dismutase (SOD) isoenzymes mRNA levels in the brain.(Original)

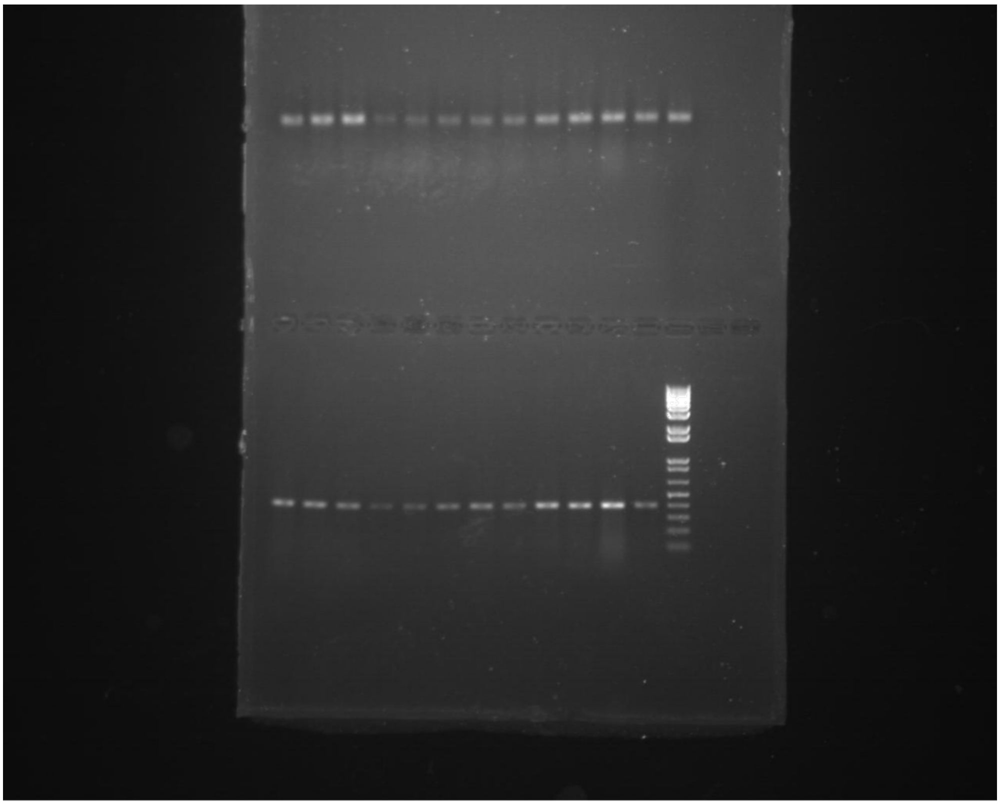

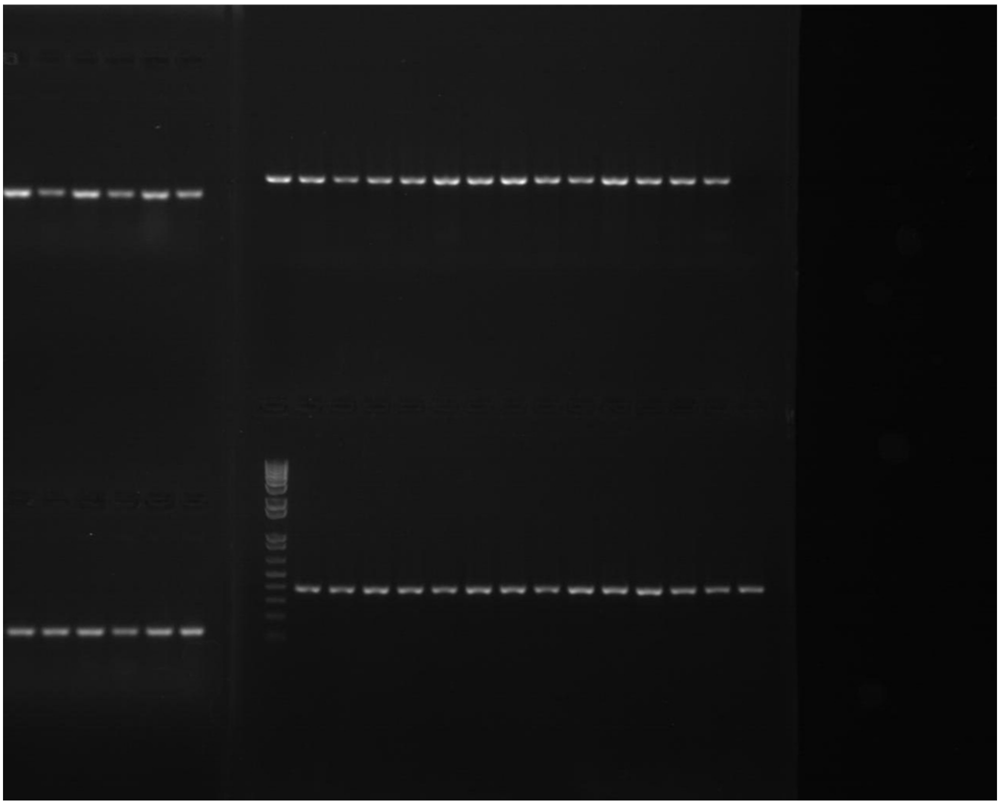

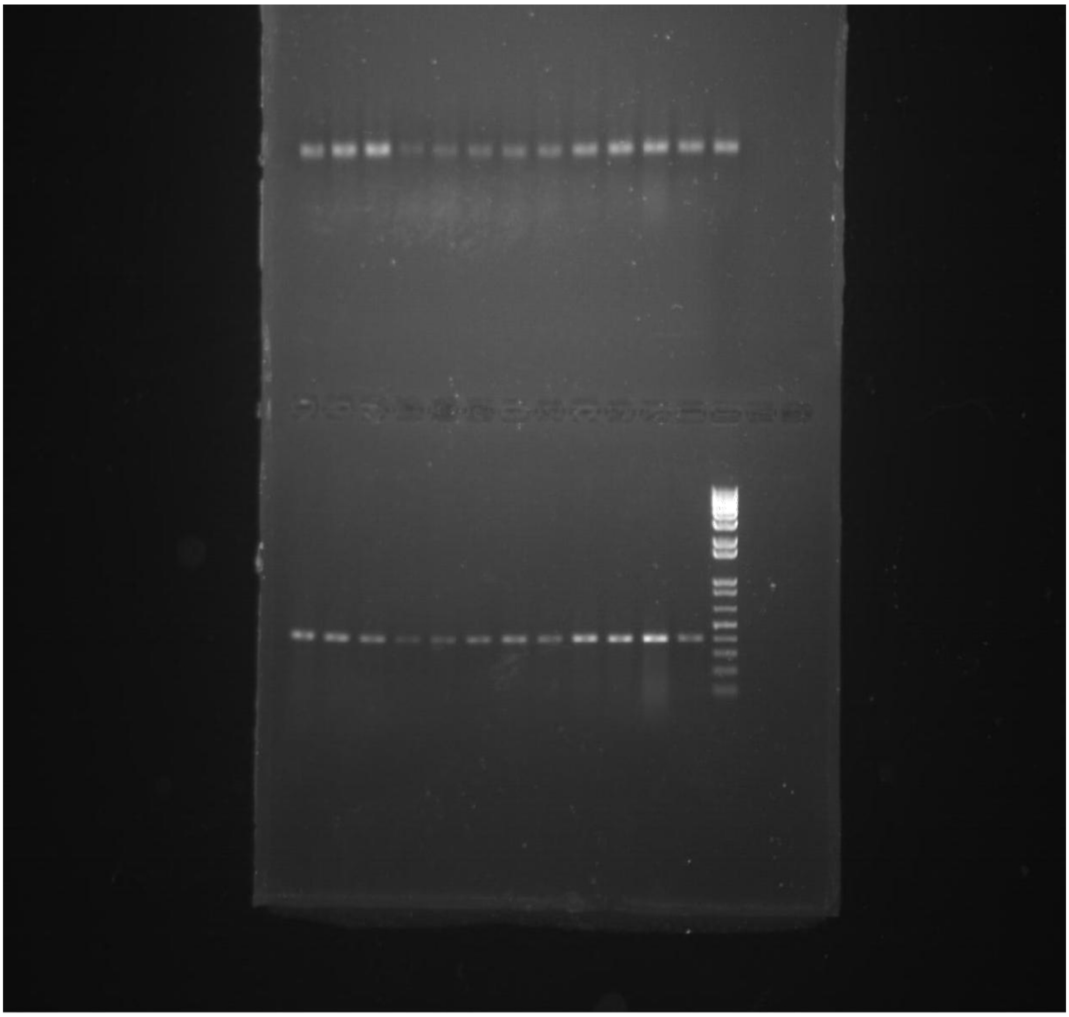

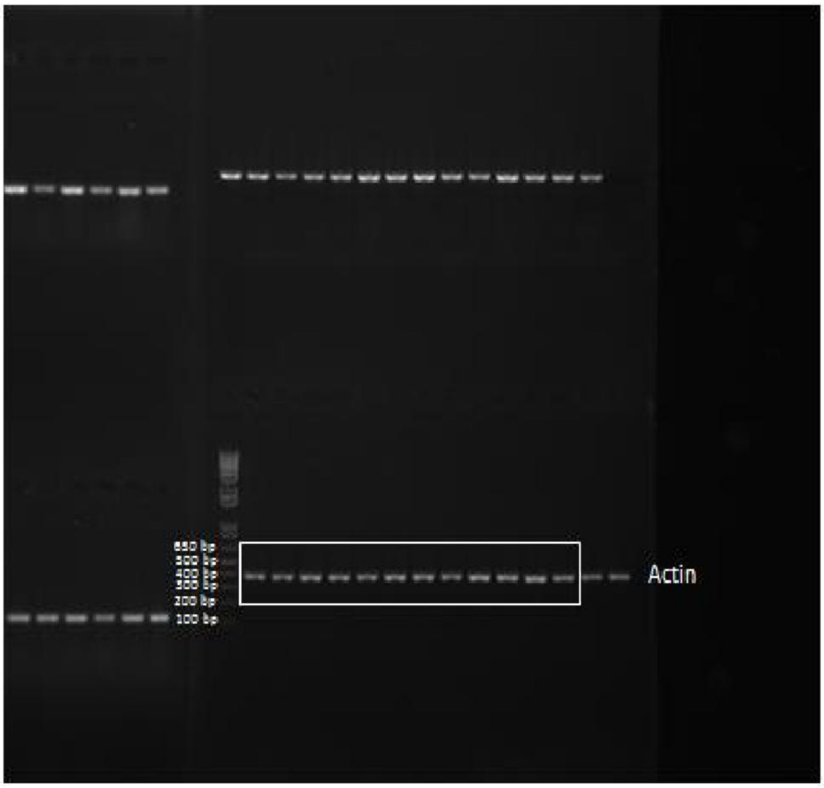
650 bp
500 bp
400 bp
300 bp
200 bp
100 bp
Actin
